# Escitalopram Induced Torsade de Pointes and Cardiac Arrest in a Patient With Surgically Treated Mitral Valve Prolapse

**DOI:** 10.7759/cureus.11960

**Published:** 2020-12-07

**Authors:** Sundeep Kumar, Jovan A Gayle, Akhil Mogalapalli, Sayed T Hussain, Analia Castiglioni

**Affiliations:** 1 Cardiovascular Disease, Saint Louis University Hospital, St. Louis, USA; 2 Internal Medicine, University of Central Florida College of Medicine, Orlando, USA; 3 Internal Medicine, University Hospitals Cleveland Medical Center, Cleveland, USA; 4 Cardiology, University of Central Florida College of Medicine, Orlando, USA

**Keywords:** out-of-hospital cardiac arrest, torsade de pointes, major depressive disorder, prolonged qtc interval, ssri

## Abstract

A 54-year-old female, with a history of prosthetic mitral valve replacement due to mitral valve prolapse one year prior, was admitted after suffering a cardiopulmonary arrest. Her initial rhythm demonstrated Torsade de Pointes with the initial electrocardiogram (ECG) showing a prolonged QT interval. Laboratory test results were normal including potassium and magnesium serum levels, and imaging did not show significant abnormalities. A review of patients' medicines showed that the patient started taking escitalopram one month prior to the presentation for major depressive disorder. Selective serotonin reuptake inhibitors (SSRI) are widely prescribed and continue to be a mainstay of treatment for multiple psychiatric conditions. It is important to keep the potential cardiovascular side effects of SSRIs in mind when prescribing. Consideration of underlying cardiac conditions is vital to decrease the likelihood of poor outcomes.

## Introduction

Selective serotonin reuptake inhibitors (SSRIs) are a class of medications widely used in primary care for a variety of common disorders such as depression, anxiety, post-traumatic stress disorder, and chronic pain. QTc prolongation is a well-documented side effect of this class [1], with Torsade de Pointes (TdP) being less common [2]. Data on escitalopram is limited, with no demonstrated correlation between plasma level and length of QT interval [3].

Structural heart disease increases the risk of developing an arrhythmia; the presence of mitral valve prolapse (MVP) is associated with an increased risk of ventricular arrhythmias and sudden death. Arrhythmias have been known to persist even after the replacement of the defective valve [4]. Although MVP is known to be arrhythmogenic [5], there is often a trigger that sets off the arrhythmogenic cascade. We present a patient with a history of longstanding mitral valve prolapse one-year post repair. He was on escitalopram and presented with arrest due to TdP. In this patient, escitalopram likely was the provoking factor for arrhythmia. Primary care physicians should remember QTc prolongation as a potentially fatal side effect of SSRIs, particularly in patients with a history of structural heart disease, and actively monitor ECG pre and post-treatment initiation.

## Case presentation

**Figure 1:**
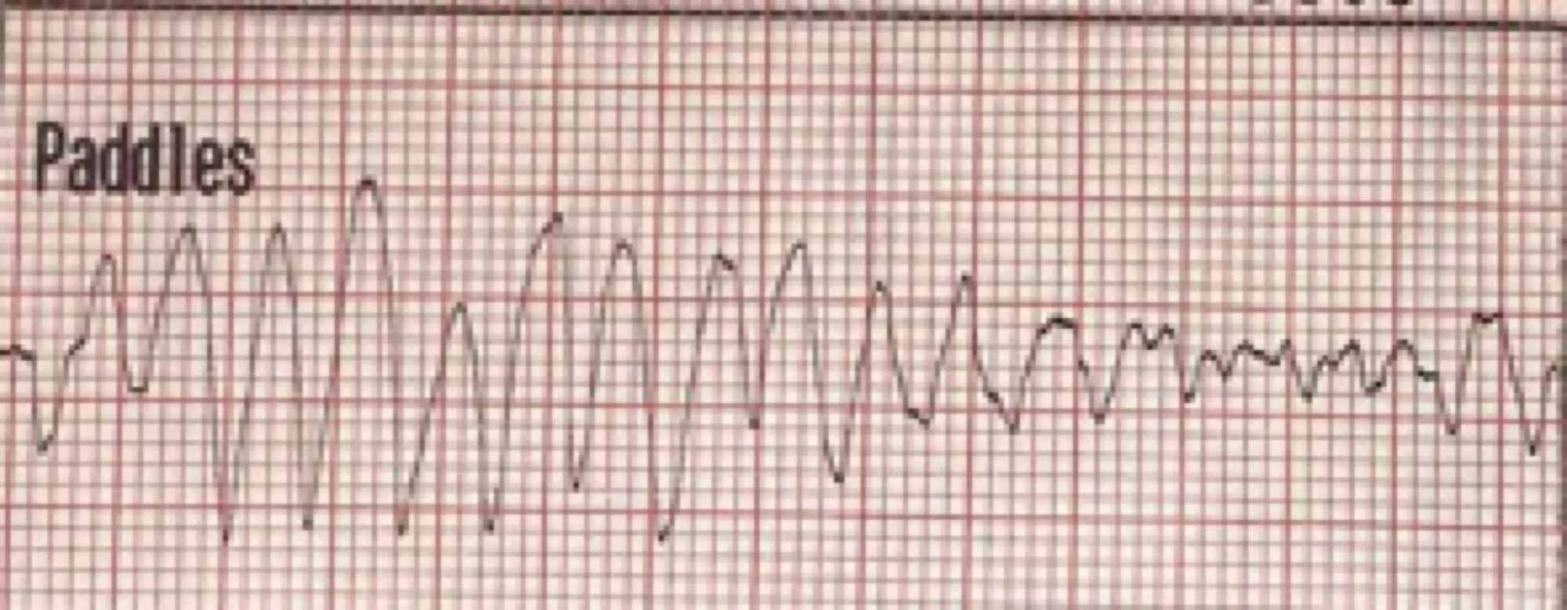
Initial Rhythm: Torsade de Pointes

**Figure 2:**
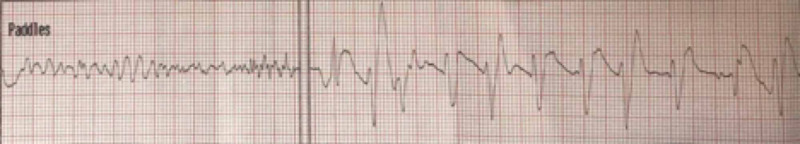
Torsade de Pointes transforming to ventricular fibrillation, followed by successful defibrillation

**Figure 3:**
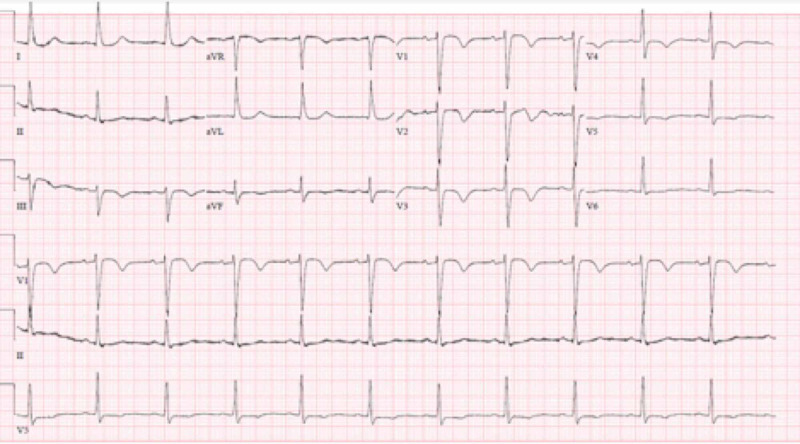
Post resuscitation 12 Lead EKG showing QTc prolongation

A 54-year-old female, with a history of prosthetic mitral valve replacement due to mitral valve prolapse one year prior, was admitted after suffering a cardiopulmonary arrest. Cardiopulmonary resuscitation was initiated by bystanders and continued by emergency medical services (EMS). The initial recorded rhythm was Torsade de Pointes which degenerated to coarse ventricular fibrillation (Figure 1), requiring multiple defibrillation shocks before the restoration of sinus rhythm and return of spontaneous circulation (Figure 2). Initial 12 lead ECG showed sinus rhythm with prolonged QTc (Figure 3). Transthoracic echocardiogram showed an ejection fraction of 40-45% with a well functioning prosthetic mitral valve. Laboratory test results demonstrated normal serum electrolyte levels, including potassium and magnesium. A detailed medical and medication history was performed after patient achieved neurological recovery. The patient confirmed that she had been taking escitalopram for a month preceding the cardiac arrest for major depressive and generalized anxiety disorders. Review of her previous records revealed an ECG that was obtained prior to starting escitalopram showing normal QTc interval. 

## Discussion

**Figure 4:**
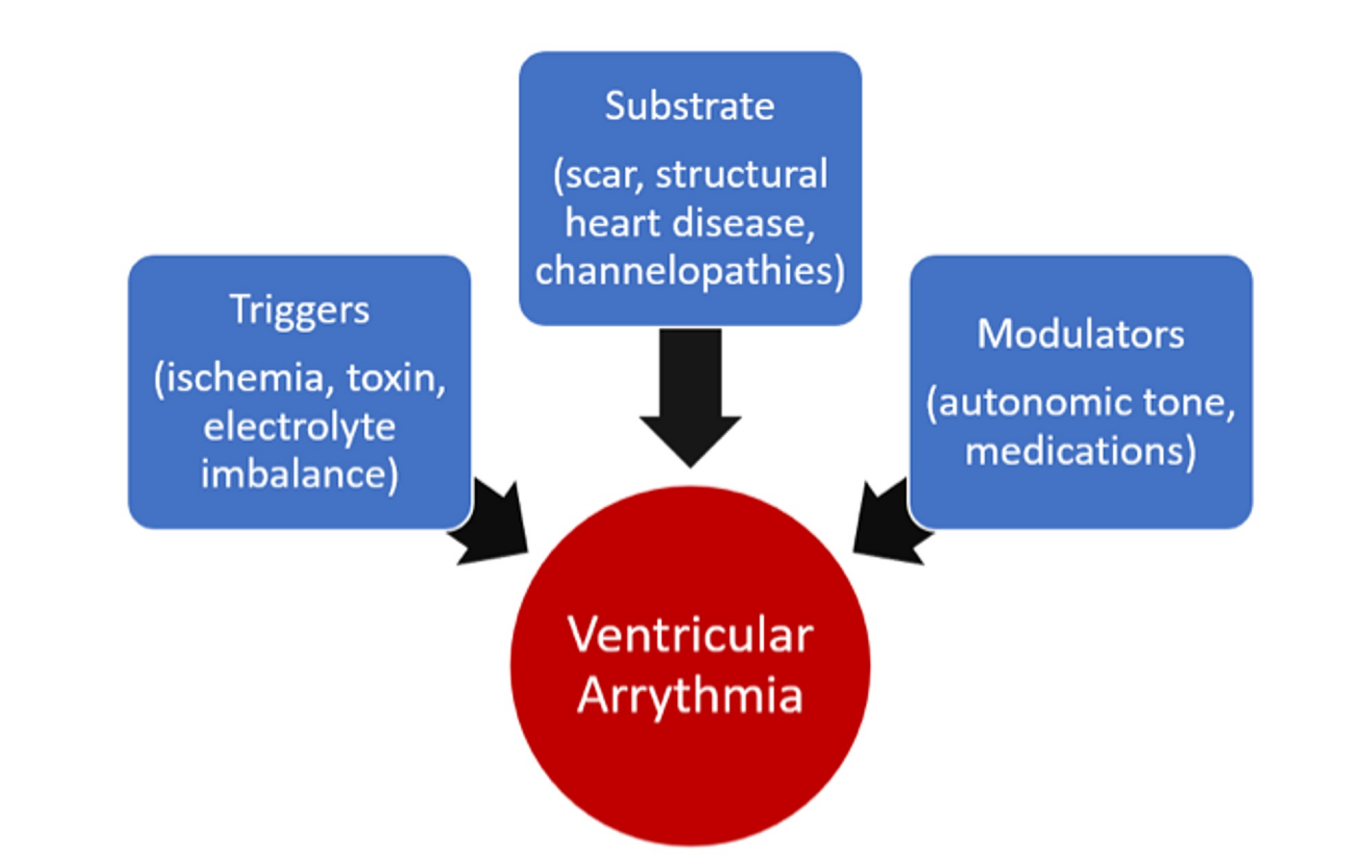
Mechanism of Arrhythmia Development

SSRIs are commonly selected for the treatment of depression due to their efficacy and wide therapeutic index [6]. Though they are known to be associated with QTc prolongation, the incidence of significant arrhythmias is low and often occurs in patients with predisposing factors such electrolyte abnormalities, congenital long QTc syndrome, and other structural cardiac diseases [7]. The pathophysiology of ventricular arrhythmias can be explained using the concept of an arrhythmogenic substrate that comprises a focal area that has the potential to generate abnormal electrical impulses, the triggers, and the modulators (Figure 4) [8]. Mitral valve prolapse (MVP) is often associated with a mechanical myocardial and papillary muscle injury and fibrosis that can form a focus from which aberrant electrical potentials can be generated [9]. Furthermore, Naksuk et al showed that mitral valve surgery did not necessarily decrease the burden of ventricular arrhythmias and proposed that the arrhythmogenic substrate formed following longstanding MVP is frequently not reversed by valve replacement surgery [4]. The ECG taken prior to surgical repair in our case, showed frequent ventricular ectopic beats in a pattern of ventricular bigeminy, hinting to the development of an arrhythmogenic focus associated with long term mechanical strain related to MVP.

The relationship between QTc prolongation, Torsade de Pointes, and ventricular fibrillation has been well established [10]. SSRIs, as a class, are known to cause QT prolongation, but only rarely cause TdP [11]. Escitalopram is an S-enantiomer of citalopram and the QTc prolongation effect of citalopram has largely been attributed to its metabolite didemethylcitalopram (DDCT) [12]. The mechanism of the QT prolongation involves the blocking of the inward potassium rectifier (IKr) current, prolonging phase 3 of cardiac repolarization and thus prolonging the QTc. If during this interval, there is repolarization originating from a different source, the compounding effect can lead to oscillating currents precipitating TdP. Thus, the QT-prolonging effect of escitalopram serves as the trigger to induce TdP in the susceptible area of the myocardium. In this case, the presence of an arrhythmic substrate from mitral valve prolapse and QTc prolongation from escitalopram are the factors that potentially acted synergistically to precipitate that ominous rhythm.

There are no clear guidelines regarding electrocardiographic monitoring in patients taking SSRIs, likely due to the relative rarity of severe arrhythmias. QTc prolongation and TdP related to SSRI are infrequent and sporadic and are usually associated with toxic drug levels or drug overdose excluding people who are at higher risk [13]. A baseline ECG is usually done before initiation of antidepressant therapy with a tricyclic anti-depressant and this has been extended to SSRIs [14].

## Conclusions

This case serves to highlight the danger of using QTc prolonging agents in patients with a history of structural heart disease such as mitral valve prolapse, even after corrective repair. It is imperative that physicians be aware of the adverse cardiovascular effects of the SSRI including QTc prolongation and TdP. In our opinion, an initial EKG is warranted before prescribing SSRIs in the presence of structural heart disease including MVP. We feel that surveillance ECGs should be obtained when increasing medication dosage. Patients should also be educated regarding the potential adverse effects of the medication class. 
